# The Practical Consideration of Poliovirus as an Oncolytic Virotherapy

**DOI:** 10.3844/ajvsp.2016.1.7

**Published:** 2016-03-03

**Authors:** Elizabeth Denniston, Hannah Crewdson, Nicole Rucinsky, Andrew Stegman, Diana Remenar, Katherine Moio, Brianne Clark, Alexandra Higginbotham, Ross Keffer, Sarah Brammer, Joseph Horzempa

**Affiliations:** 1 Department of Graduate Health Sciences, West Liberty University, West Liberty, WV, USA; 2 Department of Natural Sciences and Mathematics, West Liberty University, West Liberty, WV, USA

**Keywords:** Oncolytic Virology, Poliovirus, PVSRIPO, Glioblastoma

## Abstract

The inauguration of novel treatment strategies into the clinical setting faces a number of hurdles. In addition to treatment efficacy and safety, acceptance by doctors and patients is paramount to the success of novel therapies. Although viruses are the cause of numerous infectious diseases, these acellular entities have been harnessed over the years to benefit mankind. Recently, a recombinant Poliovirus-Rhinovirus Chimera (PVSRIPO) has shown promise for the treatment of glioblastoma in clinical trials as well as other cancer types in animal models. In this literature review, we discuss the use of PVSRIPO as an oncolytic virotherapy. In addition to being a potential treatment for glioblastoma, this recombinant virus could possibly be used against other cancers because many tumor cells express the PVSRIPO receptor antigens (CD155) and have a limited ability to control viral replication. Moreover, virus-induced immune responses contribute to the efficacy of PVSRIPO. Given the current trajectory of this experimental therapy, the possibility exists that PVSRIPO will soon be a viable treatment option for various cancer types. While many healthcare providers and cancer patients likely welcome this new viral based treatment, history has taught us that some may be skeptical and avoid its use because of the viral composition of this therapy.

## Introduction

### Risks and Benefits of Current Cancer Treatments

Current cancer treatment is dominated by chemotherapy, radiation therapy and surgery. Cancer treatments are directed to target mild cancers, or to prolong the survival time and preserve the quality of life in patients with advanced disease (Chan and McFadden, 2014; Siegel *et al*., 2012). Overall, 5-year cancer survival rates have increased over the past 40 years (Siegel *et al*., 2012). For instance, breast cancer survival rates have increased from 75.1 to 90%, childhood cancer survival rates have increased from 58.1 to 82.5% and Hodgkin’s Lymphoma survival rates have increased from 72.0 to 86.3% (Siegel *et al*., 2012).

Chemotherapy uses cytotoxic agents most often given intravenously to slow growth or kill rapidly dividing cells. While rapid division is a characteristic of cancer cells, other cells in the human body, including those in the bone marrow, gastrointestinal system and hair follicles also divide rapidly. These noncancerous mitotic cells are therefore often casualties of anticancer chemotherapies (Sugerman, 2013). Due to this collateral damage, common side effects associated with chemotherapy include reduced bone density, cognitive deficits including change in concentration, memory and mental speed and fatigue (Siegel *et al*., 2012). Other side effects include infertility, impaired pulmonary function (Siegel *et al*., 2012), nausea and vomiting (Rapoport *et al*., 2015), peripheral neuropathy and pain (Reyes-Gibby *et al*., 2015; Siegel *et al*., 2012), neutropenia and associated immunosuppression (Hashiguchi *et al*., 2015), cardiotoxicity (Siegel *et al*., 2012) and sexual dysfunction.

Radiation therapy and surgery target cancer tissue more specifically. Radiation therapy uses different types of high-energy radiation delivered either externally via a machine or internally via a radioactive material to kill target cancer cells by initiating DNA damage. This damage prevents the cell from replicating effectively which eventually causes cell death. This type of treatment can also damage cells in the surrounding area (Baskar *et al*., 2012). Common side effects of radiation therapy include diarrhea, fatigue, mucositis, skin toxicity and xerostomia (Stubbe and Valero, 2013).

Surgery as a form of treatment is even more localized and aims to remove the cancerous tissue directly. While this is the most confined of the treatments, surgery is also the most invasive (Chi *et al*., 2014; Goldfarb *et al*., 2010). Surgical treatment complications include lymphedema, pain, bowel complications, incontinence, sexual dysfunction and impaired lung function (Siegel *et al*., 2012).

More recently, recombinant virotherapy has emerged as an alternative to standard anti-cancer therapies. The objective of this literature review is to collect relevant information regarding the innovative process of virotherapy development, the current application of this therapy and the possibility of future use. Relevant articles were retrieved from the PubMed database. The novelty of this topic limited us to review those articles that were published between 2002 and the date of the submission of this manuscript. References lists were also reviewed to retrieve supplementary articles. Included articles encompass both primary and secondary literature.

Despite advances in chemotherapy, radiation and surgery, cancer continues to be a top cause of death worldwide. Therefore, it is appropriate for the healthcare community to explore diverse options of cancer treatment. The use of oncolytic virotherapy shows encouraging results for applicable clinical use in cancer treatment for future cancer patients.

## Main Text

### A Brief History of Oncolytic Virotherapy

The linkage of viral discovery to therapeutic treatment began in 1904, when Dr. George Dock hypothesized that viruses could be beneficial (Larson *et al*., 2015). This premise was founded on a case report of a female leukemia patient who entered a period of remission after undergoing an active infection with the influenza virus (Larson *et al*., 2015). In the 1950’s, additional case reports amassed, including a vignette about a cervical cancer patient who showed tumor size reduction after being injected with an attenuated rabies virus as a precautionary treatment for a dog bite (Larson *et al*., 2015). These case reports initiated clinical trials with attenuated viruses for the purpose of cancer treatment. A variety of viruses were used in these trials, including adenoviruses, hepatitis, yellow fever and West Nile, among others, but since the standards of clinical trials are not comparable to those of scientific research today the success of these clinical trials is difficult to ascertain (Larson *et al*., 2015). As technology continued to progress, specifically genetic engineering, the possibility exists for an array of viruses to be manipulated and subsequently adapted for clinical use (Larson *et al*., 2015).

### The Premise of Oncolytic Virotherapy

The practice of oncolytic virotherapy includes selecting a virus that meets defined criteria for safety and efficacy in humans. First, the virus must exhibit selectivity for cancer cells, while avoiding normally functioning human cells. Some viruses have the innate ability to target cancer cells, such as human orphan viruses (viruses not associated with disease) and animal viruses that lack specificity for human cells, like the myxoma virus (Goetz and Gromeier, 2010). Other viruses, including poliovirus, adenovirus and herpes simplex virus, have the natural ability to target cancer cells, but must be genetically modified to remove virulence potential and protect the safety of the host (Goetz and Gromeier, 2010). For oncolytic polioviruses, this was accomplished in part by replacing an intrinsic genetic component critical for viral protein translation (IRES element) with an equivalent component of human rhinovirus type 2. This change prohibited viral replication in normal neuronal cells, but supported replication in tumors of the central nervous system and elsewhere (Goetz and Gromeier, 2010).

Secondly, the recombinant virus must be genetically stable and be incapable of reverting back to its wild-type form while replicating inside tumor cells (Brown *et al*., 2014). In addition, the genetically modified virus must effectively destroy tumor cells and evade or remain functional during immune responses (Brown *et al*., 2014). Oncolytic viruses must be able to bind to and infect target cancer cells. The poliovirus naturally binds to CD155 (Necl-5, nectin-like molecule 5) which is expressed in abundance on many malignant cancer cells (Brown *et al*., 2014). Virotherapies have been administered in a number of routes experimentally over the years (Southam, 1960). Recent studies with recombinant polioviruses indicate that direct intratumoral administration is promising. After administration and viral entry, replication ensues within the cancerous cells which leads to tumor cell lysis and death (Goetz and Gromeier, 2010; Brown *et al*., 2014). Newly replicated viruses freely invade additional local tumor cells and can circulate to metastatic cells elsewhere in the body, repeating the replication-lysis process (Sze *et al*., 2013).

The host immune response is critical for the success of oncolytic virotherapies (Brown *et al*., 2014). The type 1 interferons, INFA and B, are cytokines produced in response to viral infections (Brown *et al*., 2014). These molecules normally lead to a cascade of events aimed to limiting viral replication. However, antiviral responses are often inadequate in cancerous cells, limiting the effectiveness of these cytokines to initiate viral clearance (Brown *et al*., 2014). Conversely, type 1 interferons are important for the development of anti-cancer adaptive immunity, a scenario that benefits the efficacy of oncolytic virotherapies (Brown *et al*., 2014).

### Normal Infectious Processes of Polioviruses

The poliovirus is a small, single stranded RNA virus with an icosahedral protein coat that causes disease in both animals and humans (Kotla and Gustin, 2015). This virus is inhaled through the nasal and pharyngeal passageways and multiplies rapidly in the epithelial cells within the pharynx and GI tract. Infection is initiated upon viral attachment of capsid proteins VP1, VP2 and VP3 to CD155 receptors on host cells. Subtle conformational changes take place within the major viral capsid proteins (VP1, VP2 and VP3) that allow the virus to attach to the membrane, create a pore and release the viral RNA into the cytoplasm. The viral RNA is then replicated and translated to synthesize viral proteins within the host cell. Translation results in a polyprotein that is then configured into multiple intermediate forms before ultimately producing the infectious virus form known as a virion. These viral proteins also produce another capsid for the virus to exit and infect nearby host cells as a virion (Hogle, 2002).

Viral RNA is recognized in infected cells by receptors both on the membrane and in the cytoplasm of the host cells. These receptors include TLRs and RIG-like receptors, RIG-I and MDA-5 (Hogle, 2002). Once viral RNA is recognized via RIG-like receptors, cellular signaling leads to activation of specific transcription factors, one being IRF-3 which activates the type I interferon response. The activation of the type I interferon response play a key role in slowing disease progression and spread. Poliovirus contains many mechanisms to inhibit this type I interferon response. For instance, poliovirus blocks the activation of IRF-3 thereby inhibiting the type I interferon response. Studies suggest the virus modulates stress granules formed in cellular cytoplasm in response to a viral infection which in turn may contribute to blocking IRF-3 activation (Kotla and Gustin, 2015).

### Mechanism and Therapeutic Benefit of PVSRIPO

Through genetic manipulation, polioviruses have been engineered to be highly attenuated to ensure safety and proper oncolytic function (Goetz *et al*., 2011). One such recombinant poliovirus, the polio: Rhinovirus chimera (PVSRIPO) has shown promise for glioblastoma treatment. To attenuate PVSRIPO, the Internal Ribosomal Entry Site (IRES) of the PVSRIPO was substituted with the IRES from Human Rhinovirus type 2 (HRV2), which is an enterovirus specific to the respiratory system. The IRES is a nucleotide sequence located within the middle segment of messenger RNA (mRNA) used to initiate translation. These IRES elements are features paramount to viral protein synthesis. Changing the IRES of the PVSRIPO allows this recombinant virus to replicate in transformed or cancerous cells. However, the HRV2 IRES precludes replication in normal neurons, preventing adverse neural effects, but still rendering the PVSRIPO cytotoxic to cancer cells (Goetz and Gromeier, 2010; Brown and Gromier, 2015; Goetz *et al*., 2011). Specifically, attenuation of the IRES causes an inability of the PVSRIPO mRNA to recruit eukaryotic Initiation Factor 4G (eIF4G). Lack of eIF4G recruitment, which is a cap-binding protein that bridges ribosomal subunits to initiate protein synthesis, prevents viral protein translation which causes the attenuated PVSRIPO to be non-cytolytic in the central nervous system cells of the host (Goetz *et al*., 2011). This attenuation of PVSRIPO in normal host cells allows for the specificity of cytotoxicity in cancerous tissue.

Many attributes of PVSRIPO make this virus capable of lysing cancerous cells but incapable of infecting and replicating in non-malignant host cells (Brown and Gromier, 2015). PVSRIPO has a natural affinity for CD155 antigens, which are specific surface markers abundant on glioblastoma cells. CD155 is also expressed on antigen presenting cells such as dendritic cells and macrophages (Brown *et al*., 2014). Unlike cancer cells that have a weakened antiviral response, macrophages and dendritic cells exposed to PVSRIPO are capable of limiting viral replication and can engage T cells (Brown *et al*., 2014). PVSRIPO has potential to be used as a treatment against a multitude of cancers as many diverse tumor cells exhibit abundant surface expression of CD155 (Brown *et al*., 2014; Merrill *et al*., 2004). The PVSRIPO affinity for CD155 coupled with the attenuation of this virus- which blocks the viral cytotoxicity in normal host cells- are central to the selectivity of cancerous tissue by this virotherapy.

Many cancers can suppress immune responses, thereby prolonging the longevity of the associated disease. PVSRIPO has the ability to activate the host innate immune response within tumor cells, even those that elicit immunosuppression (Brown and Gromier, 2015). In addition to viral molecules activating type I interferons, this is also accomplished by the PVSRIPO-induced lysis of tumor cells which leads to the release of Danger-Associated Molecular Patterns (DAMPs) that activate inflammatory responses. Moreover, tumor cell lysis results in the exposure of tumor antigens to antigen presenting cells and subsequently the adaptive immune response (Brown *et al*., 2014). These adaptive responses include activation of NK and Th1 cells, which target and destroy the tumor cells (Goetz *et al*., 2011).

### Current Application

Oncolytic virotherapy using PVSRIPO has been experimentally successful in targeting glioblastoma and neuroblastoma cells, but has been ineffective in the treatment of Burkitt’s Lymphoma (Toyoda *et al*., 2007; Gromeier *et al*., 2014). The use of polioviruses to treat neuroblastoma in mice yielded favorable results, identifying the possibility for clinical application for similar types of cancers, especially those prominent in childhood. Neuroblastoma is one of the most frequent cancers in children and treatment is invasive and of high risk with poor outcomes, so the use of PVSRIPO could be a significant new treatment option (Toyoda *et al*., 2007).

Currently, several studies using mice suggested that PVSRIPO is an effective treatment methodology. In one study, subcutaneous implantation of neuroblastoma followed by direct tumoral administration of attenuated poliovirus resulted in complete eradication of the tumor. Immunological analyses indicated that this tumor was eliminated, in part, by CD8(+) T cells (Toyoda *et al*., 2011). In another study, twelve athymic mice were implanted with glioma xenographs *in vivo*. After a period of time to allow for tumor growth, PVSRIPO inoculation was administered. Ten days after PVSRIPO inoculation, the median tumor size had shrunk by approximately 45%. After another 18 days, tumors were destroyed in these mice indicating that even in the absence of T cells, the PVSRIPO is effective in eliminating tumor cells (Dobrikova *et al*., 2008). Phase I clinical trials for treatment of human glioblastoma by PVSRIPO are currently in progress with continued patient recruitment at Duke University (Gromeier *et al*., 2014). In this clinical trial, upon biopsy confirmation of a viable tumor, a catheter is implanted into the tumor that directly delivers the PVSRIPO suspension to the glioblastoma. The catheter is then removed immediately after inoculation and the patients are radiographically evaluated after treatment for tumor changes (Friedman, 2000). PVSRIPO induces a lethal and irreversible virolytic effect and host immune response cytotoxic to the tumor as early as one hour post administration and favorable outcomes have thus far been recorded as many as 20 months after treatment (Gromeier *et al*., 2014). Should the results of this trial remain positive, further clinical trials using PVSRIPO to treat other types of cancer are warranted.

### Risks and Benefits of Oncolytic Virotherapy

Oncolytic virotherapy as a treatment is in its infancy. Therefore, factors regarding the overall safety and optimization of this therapy have not yet been fully vetted. Relative to the current standard of care, virotherapy is much less invasive and debilitating (Fong, 2015). Moreover, this form of oncolytic therapy is highly selective. Injections can be administered locally, which ensure an increased uptake by tumor cells. The viral machinery has been engineered to allow for selection and replication within tumor cells, limiting infection of non-cancer cells of the patient. The poliovirus itself has added benefits (Fong, 2015). The poliovirus has a large RNA size capability making this virus amenable to genetic manipulations. Virotherapies such as PVSRIPO have the ability to specifically target cancer cells and efficiently induce lysis (David *et al*., 2009). However, this efficiency necessitates the development of patient selection criteria. Currently, there is no standard dose for virotherapy as certain viruses and tumors require different dosage paradigms (David *et al*., 2009). Ultimately, a dosing strategy will be created that maximizes effectiveness and minimizes risk (Fong, 2015). The optimization of viral production will also be a challenge; little information about cost is currently available. Hospitals will also face challenges with administration, transport, storage and disposal of these viral agents. Although these viruses are attenuated, they pose a potential threat to immuno-compromised patients. Many challenges lie ahead involving the potential implementation of these treatments. However, PVSRIPO serves as a promising treatment option for the future of cancer therapy and may usher in a wave of virus-based therapeutics (Fong, 2015).

### Significance

According to the Centers for Disease Control and Prevention (CDC) National Program of Cancer Registries (NPCR) most recent report of national cancer trends, 426.6 of every 100,000 of people who obtained medical attention in 2010 registered as a cancer patient28 These cancers included types such as prostate cancers, lung cancers, leukemia, esophageal and stomach cancers, to breast and lymphatic cancers (Edwards *et al*., 2014). The number of patients who have cancer in the United states, in addition to the broad spectrum of body systems that cancer can affect, impresses the importance of exploring safe and effective new treatments of all cancers. Additionally, the process of a cell becoming malignant is dynamic and unique, much like the patients who will be receiving treatment. Therefore, treatment should be able to meet the demands of the dynamic process of cancer development without harming the healthy cells within the host.

Cancers arise from an accumulation of genetic mutations that transform a healthy cell into one that causes disease. Because cancer cells are nearly identical to healthy cells, selective toxicity with current standard therapies is difficult to attain. Further, difficulty arises from the inability to determine the specific changes the cancers undergo to create malignancy (Workenhe and Mossman, 2014). Due to the rapid ability of a virus to be changed by recombinant RNA technology, researchers may be able to make “designer viruses” specific to a patient's treatment needs (Parato *et al*., 2005). Viruses can be modified to invade cancer cells depending on status and speed of growth, receptor type displayed on cancer cell surface, or transcriptional and translational pathway targets within the specific cancer type (Parato *et al*., 2005). By targeting these specific mechanisms, viruses can potentially be designed to target the unique process that culminate in malignancy. Recombinant genetic technology may allow oncologists to create an oncolytic virus that targets the specific cancer cell type and location. A virus’ propensity to replicate within a host cell and to target cells specifically provides oncolytic virotherapy a pharmacokinetic advantage over conventional cancer therapies (Parato *et al*., 2005).

As previously mentioned, oncolytic viruses have the ability to stimulate the patient’s immune system while replicating within the tumor cells. Replication within the tumor cells leads to a recruitment of immune cells to this area. In addition to enhancing the cancer fighting ability of the body, this immune activation may benefit patients that have developed immuno-tolerance to their current standard of care chemotherapeutic (Workenhe and Mossman, 2014). Continued use of chemotherapies can lead to cancer cells that are drug resistant or tolerant to these conventional therapies (Workenhe and Mossman, 2014). By using a viral modality to trigger cancer death which involves activation of the host immune response in combination with the use of conventional cancer-targeting treatment, drug resistance and tolerance may be limited (Workenhe and Mossman, 2014).

### Clinical Perspective

Given the current progress of the experimental and clinical phases of PVSRIPO therapy, the possibility exists that this recombinant virus will soon be a viable treatment option for glioblastoma and various other cancer types. While many healthcare providers and cancer patients may embrace this new virotherapy, history suggests that some may be skeptical and avoid the use because of the viral composition of this treatment. For instance, attenuated viruses have been used safely for decades to vaccinate individuals preventing countless cases of polio, mumps, measles, rubella and other viral diseases. However, due to a fraudulent study and a media explosion, some individuals falsely believe that vaccines cause autism. We suspect that cancer patients and even some physicians may be skeptical of PVSRIPO due to the viral nature of this therapy. Then again, the imminent threat of cancer may offset any fears. Patient education efforts and physician outreach may be necessary to establish virotherapy as a safe cancer treatment option.

The very first oncolytic virotherapy has just recently been approved by the FDA (Greig, 2016). This therapy-talimogene laherparepvec (Imlygic) can be used to treat patients with skin and lymph node melanoma lesions (Greig, 2016). Talimogene laherparepvec is comprised of an attenuated herpes virus that causes cancer cells to produce GM-CSF, a potent cytokine that acts as a growth factor for white blood cells and induces differentiation of monocytes. Lessons learned from the use of talimogene laherparepvec will undoubtedly contribute to the education efforts and implementation of forthcoming virotherapies such as PVSRIPO.

Based on a cohort study of FDA trends of expedited drug development and approval programs, the novelty and aforementioned advantages of PVSRIPO indicate that this treatment has the potential to be approved for patient use (Kesselheim *et al*., 2015). Pending FDA approval, physicians will have the option to implement this novel form of therapy for their patients. As with all initiative therapies, the physician needs to play an active role in patient education. The patient should be informed of the mechanism of action of this form of therapy to fully understand the oncolytic role PVSRIPO will play in the treatment of the patient’s cancer. Additionally, all potential adverse reactions should be fully disclosed to all potential patients. Although evidence suggests that PVSRIPO has the potential to provide great therapeutic benefit to cancer patients, we hypothesize that use in clinical practice will rely heavily on physician acceptance and patient education.
